# Dynamic Foraging Behavior Performance Is Not Affected by *Scn2a* Haploinsufficiency

**DOI:** 10.1523/ENEURO.0367-23.2023

**Published:** 2023-12-08

**Authors:** Selin Schamiloglu, Hao Wu, Mingkang Zhou, Alex C. Kwan, Kevin J. Bender

**Affiliations:** 1 Neuroscience Graduate Program, University of California, San Francisco, CA 94158; 2 Center for Integrative Neuroscience, Department of Neurology, University of California, San Francisco, CA 94158; 3 Interdepartmental Neuroscience Program, Yale University School of Medicine, New Haven, CT 06511; 4 Meinig School of Biomedical Engineering, Cornell University, Ithaca, NY 14853

**Keywords:** autism spectrum disorder, Scn2a

## Abstract

Dysfunction in the gene *SCN2A*, which encodes the voltage-gated sodium channel Na_v_1.2, is strongly associated with neurodevelopmental disorders including autism spectrum disorder and intellectual disability (ASD/ID). This dysfunction typically manifests in these disorders as a haploinsufficiency, where loss of one copy of a gene cannot be compensated for by the other allele. *Scn2a* haploinsufficiency affects a range of cells and circuits across the brain, including associative neocortical circuits that are important for cognitive flexibility and decision-making behaviors. Here, we tested whether *Scn2a* haploinsufficiency has any effect on a dynamic foraging task that engages such circuits. *Scn2a*^+/−^ mice and wild-type (WT) littermates were trained on a choice behavior where the probability of reward between two options varied dynamically across trials and where the location of the high reward underwent uncued reversals. Despite impairments in *Scn2a*-related neuronal excitability, we found that both male and female *Scn2a*^+/−^ mice performed these tasks as well as wild-type littermates, with no behavioral difference across genotypes in learning or performance parameters. Varying the number of trials between reversals or probabilities of receiving reward did not result in an observable behavioral difference, either. These data suggest that, despite heterozygous loss of *Scn2a*, mice can perform relatively complex foraging tasks that make use of higher-order neuronal circuits.

## Significance Statement

Deleterious variation in the *SCN2A* gene is associated with neurodevelopmental disorders. As such, considerable resources have been devoted to understanding the cellular and behavioral changes underlying *Scn2a* haploinsufficiency. Previous work showed that excitatory neurons in prefrontal cortex (PFC) are strongly affected by *Scn2a* haploinsufficiency at the cellular level. *Scn2a* is also expressed in the striatum and in midbrain dopamine neurons. Given the role of these regions, as well as PFC, in behavioral flexibility, we examined a dynamic foraging task in *Scn2a*^+/−^ mice thought to engage such circuits. We observed no behavioral deficits in this task because of *Scn2a* loss, suggesting that these mice can perform complex foraging tasks despite alterations in Na_V_1.2 expression levels.

## Introduction

Deleterious mutations in the gene *SCN2A*, which encodes the voltage-gated sodium channel Na_v_1.2, constitute one of the leading risk factors for neurodevelopmental disorders, increasing the odds of developing autism spectrum disorder and intellectual disability (ASD/ID) to levels beyond those calculable from studies of large cohorts (e.g., an infinite odds-ratio; Sanders et al., 2012; Ben-Shalom et al., 2017; Satterstrom et al., 2020; Fu et al., 2022; Rolland et al., 2023). ASD-associated *SCN2A* dysfunction is typically a result of haploinsufficiency, where loss of only one gene copy cannot be compensated for by the remaining allele (Wolff et al., 2017; Ben-Shalom et al., 2017; Sanders et al., 2018; Begemann et al., 2019). *Scn2a* is expressed throughout the brain (Kang et al., 2004; GTEx Consortium, 2015). In neocortex, *Scn2a* is expressed predominantly on the plasma membranes of pyramidal cells (Hu et al., 2009; Li et al., 2014; Yamagata et al., 2017), and previous work has shown that *Scn2a* haploinsufficiency in mice results in reduced dendritic excitability in prefrontal cortical pyramidal cells (Spratt et al., 2019; Nelson et al., 2022; Tamura et al., 2022). Beyond neocortex, *Scn2a* is also expressed in striatal medium spiny neurons (Miyazaki et al., 2014), where a >50% reduction in expression can result in neuronal hyperexcitability (Zhang et al., 2021), as well as in midbrain dopamine neurons (Yang et al., 2019), where its functional role has not yet been elucidated. This expression pattern suggests that *Scn2a* haploinsufficiency might affect behaviors involving corticostriatal circuits and their modulation via midbrain dopaminergic sources.

Behavioral flexibility depends on activity in prefrontal cortex (PFC), striatum, and midbrain dopaminergic neurons (Rolls et al., 1994; Fellows and Farah, 2003; Samejima et al., 2005; Kennerley et al., 2006; Lee and Seo, 2007; Ragozzino, 2007; Lau and Glimcher, 2008; Rutledge et al., 2009; Simon et al., 2015; Hamid et al., 2016; Del Arco et al., 2017; Fiuzat et al., 2017; Ueda et al., 2017; Donahue et al., 2018; Nakayama et al., 2018; Bari et al., 2019; Brigman et al., 2013), and behavioral inflexibility and perseveration are oft-noted features of ASD (Pasciuto et al., 2015). Several mouse models of ASD, including *Fmr1* knock-out mice and a model of the human 15q11–13 duplication, have recapitulated aspects of this phenotype. Consistently across these models, transgenic animals all had impaired reversal learning, a measure of behavioral flexibility, compared with wild-type (WT) littermates in paradigms like the Morris water maze and Y-maze (D’Hooge et al., 1997; Nakatani et al., 2009; Tsai et al., 2012; Dickson et al., 2013; Huang et al., 2013; Santini et al., 2013; Jiang-Xie et al., 2014; Pasciuto et al., 2015). In contrast, *Scn2a*^+/−^ males showed only a mild impairment on reversal learning in a water T-maze task (Spratt et al., 2019).

There are other behavioral assays to measure behavioral flexibility, however. Dynamic foraging, for example, requires the animal to use prior outcomes to guide current decisions, as in the water maze, but also requires learning in an uncertain environment. Choices are not associated with specific outcomes. Rather, rewards are delivered probabilistically, and the reward probabilities can switch over time (Soltani and Izquierdo, 2019). Because the task is nontrivial and challenging, it can be applied to nonhuman primates (Donahue and Lee, 2015) and humans (Sutton and Barto, 2018) and is thus more readily translatable. Several animal ASD models had impaired reversal learning in dynamic foraging tasks, although the nature of their errors varied (Roh et al., 2020; Alvarez et al., 2023; Schmitt et al., 2023). Numerous studies have shed light onto the neural circuits involved in dynamic foraging, implicating regions including medial frontal cortex (Atilgan et al., 2022), orbitofrontal cortex (Groman et al., 2019), and dorsal striatum (Tai et al., 2012). More specifically, silencing PFC projections to dorsomedial striatum, including those originating in layer 5, increased choice bias (and thus reduced flexible choice) in two similar foraging tasks (Nakayama et al., 2018; Bari et al., 2019). Given the importance of Na_v_1.2 for dendritic integration in PFC layer 5 pyramidal cells (Spratt et al., 2019; Nelson and Bender, 2021) and its broad expression in additional circuits that support behavioral flexibility and foraging behavior, we sought to determine whether *Scn2a* haploinsufficiency had an effect on a dynamic foraging task.

Here, we show that *Scn2a*^+/−^ mice do not exhibit behavioral differences compared with WT littermates, and both groups can readily learn the task. For both deterministic and probabilistic reward contingencies, mice of both genotypes were able to adapt their choice behavior equally after a reward contingency reversal. *Scn2a*^+/−^ mice and WT littermates did not show differences in motivation as measured by self-initiated intertrial interval and also appeared to use similar strategies to obtain reward. Furthermore, varying block length or reward probabilities did not unmask a behavioral difference in *Scn2a*^+/−^ animals. There were no differences across sexes for any of the behavioral metrics analyzed. As a final note, we observed spontaneous seizures in four *Scn2a*^+/−^ animals, which may relate to housing conditions required to motivate animals to perform these behaviors. Overall, these data demonstrate that mice can perform tasks that require learning and behavioral flexibility in light of heterozygous loss of Na_V_1.2 expression.

## Materials and Methods

### Animals

All animal procedures were performed in accordance with the University of California, San Francisco and Yale University Institutional Animal Care and Use Committees. Postnatal day (P)54–P119 *Scn2a*^+/−^ mice or wild-type (WT) littermates of both sexes on the C57BL/6J background were used. *Scn2a*^+/−^ mice were originally described by Planells-Cases et al. (2000). Animals were genotyped with PCR. Mice were maintained on a 12/12 h light/dark cycle and had *ad libitum* access to food. No animals were excluded based on behavioral performance. Two of 50 animals failed to complete the entire behavioral assay. One WT mouse died during the training period for unknown reasons. One *Scn2a*^+/−^ mouse experienced a terminal seizure immediately after a training session when returned to their home cage. The experimenter was blind to the animal genotypes while running the behavior.

### Freely moving behavior

All experiments were conducted in purpose-built acrylic boxes (roughly 5′′ × 7′′, left and right poke walls were angled to form a pentagon). Each box had three custom-built pokes, each with a white LED and infrared emitters and receivers (Sparkfun). The left and right pokes dispensed a solution of 10% sucrose in water through metal tubes fitted into the poke, and the sucrose delivery was controlled with solenoid pinch valves (NResearch). The task was run using an mbed microcontroller and custom MATLAB and Statescript (Spike Gadgets) scripts, and all behavioral analyses were completed with custom MATLAB scripts.

Three days before the start of behavioral training, animals were placed on water deprivation for 24 h and on subsequent water restriction to maintain weight at 85% predeprivation weight. If insufficient sucrose solution was consumed during the behavioral session, supplemental water was given in the home cage to maintain weight.

In the first stage of training, animals learned to receive reward from the side ports by poking their noses in the central nosepoke. On days 1 and 2, animals were placed in the behavioral boxes, and self-initiated pokes into the center poke triggered automatic reward delivery (∼3 μl 10% sucrose solution) in the left (for one session) or right (for the other session, counterbalanced across animals) port. Once the animal poked its nose into the baited port, additional reward was dispensed. Animals completed 100 trials or 1.5 h of training, whichever came first, each day.

In the second stage of training, animals learned to collect reward from both side ports in a single session. Animals self-initiated trials by poking their noses into the center nosepoke. A light would turn on at the center nosepoke to signal reward availability. Reward availability was set at 100% for one side port and 0% for the other, and animals could choose between the two. A nose poke to the high reward port resulted in immediate reward delivery (∼2 μl 10% sucrose solution) at that port. The location of the high reward probability port underwent uncued reversals every 100 trials or once the animal selected the high reward port 9 of the 10 previous trials. Animals performed an average of 714 trials a day and were trained on this version of the task until they reached 80% performance 2 d in a row or after 10 sessions.

In the full version of the foraging task, animals self-initiated trials by poking their noses into the center nosepoke. Reward availability for each port was independently assigned to 60% for one port (high reward probability) versus 15% for the other (low reward probability) unless noted in the text and figures. Once reward was assigned to a port, the reward was available until the animal chose that port (Sugrue et al., 2004; Lau and Glimcher, 2008; Fonseca et al., 2015; Bari et al., 2019). The location of the high reward probability port underwent uncued reversals every 80 trials unless noted in the text and figures. Animals performed an average of 938 trials per session on this full version of the task.

### Head-fixed behavior

Before surgery, the mouse was given carprofen (5 mg/kg, s.c.; 024751, Butler Animal Health) and dexamethasone (3 mg/kg, intramuscular; 002458, Henry Schein Medical Animal Health). Anesthesia was induced with 3% isoflurane in oxygen, and lowered to 1–1.5% during surgery. The mouse was positioned in a stereotaxic apparatus (940, David Kopf Instruments) and sat on a 38°C water-circulating heating pad (Stryker Corp). The scalp was removed to expose the skull. A custom-made stainless steel headplate (eMachineShop) was glued onto the skull with C&B Metabond (Parkell). After the surgery, carprofen (5 mg/kg, s.c.) was injected each day for the following 3 d. The mouse recovered for at least 7 d after the surgery before the start of the behavioral training.

A comprehensive guide on constructing the apparatus is available at https://github.com/Kwan-Lab/behavioral-rigs. For the behavioral apparatus, a specialized lick port with two lick spouts, made from blunted 20-gauge stainless-steel needles, was positioned in front of the mouse. For controlled fluid delivery at the lick spouts, two solenoid fluid valves (MB202-V-A-3–0-L-204, Gems Sensors & Controls) were employed. Each spout’s water delivery could be independently regulated. The quantity of water dispensed per pulse was tuned to ∼4 μl by adjusting the duration of the electrical pulse administered to each valve via a second data acquisition unit (USB-201, Measurement Computing). To produce the auditory cue, a pair of speakers (S120, Logitech) was positioned in front of the mouse. For head fixation, the head plate was securely held in place using a stainless-steel holder (eMachineShop). The mouse sat within an acrylic tube (8486K433; McMaster-Carr), allowing for minor postural adjustments while restricting major movements. Contact between the tongue and the lick spouts were detected using a battery-powered electronic circuit. Signals from this circuit were transmitted to a computer via a data acquisition unit (USB-201, Measurement Computing). The captured data were logged using the Presentation software (Neurobehavioral Systems). The entire apparatus was enclosed within an audiovisual cart, the walls of which were insulated with soundproof acoustic foams (5692T49, McMaster-Carr).

Mice were fluid-restricted during behavioral training. On training days, the animal received all of its water intake from behavioral training that occurred one session per day, 6 d per week. On nontraining days and on days if its weight fell below 85% of their pretraining value, water was provided *ad libitum* in their home cage for 5 min. Before the behavioral training, the animal was handled and habituated to head fixation for increasing durations over 3 d. The training procedure involves four phases including three phases to shape the behavior before the final task phase.

During phase 0 (∼2 d), the experimenter manually administered 50 water rewards through each port (100 rewards in total), with the goal to elicit reliable licks. In cases where the animal did not readily engage in licking to consume the water rewards, the experimenter used a blunted syringe to gently guide the animal toward the spout, facilitating a licking response.

During phase 1 (∼1 d), the animal was trained to alternate between the two lick ports to receive water rewards. At the beginning of each trial, an auditory cue (5 kHz, 0.2-s-long pure tone) was played. Subsequent to cue onset, there was a 5s response window for the animal to act. The first lick within the response window is the animal’s response. The playback of the auditory cue was terminated early if a response was recorded before the entire stimulus was played. The animal was trained to alternate its choices. Specifically, if the mouse received a reward on the left side on the current trial, it must choose the right side on the next trial to trigger the next reward, and vice versa. The session would end if the animal did not lick during the response window for 20 consecutive trials.

During phase 2 (∼2 d), the animal was trained to alternate, while also having to suppress licking before the go cue. Phase 2 is the same as phase 1, with two modifications. First, the response window was shortened to 2 s. Second, a no-lick period was introduced between trials. The no-lick period began 3 s after the animal’s response. Initially, the duration of the no-lick period was determined by drawing a random number from a truncated exponential distribution (λ = 0.3333, minimum = 1, maximum = 5). If any lick was detected during the no-lick period, an additional duration drawn from the same truncated exponential distribution would be added to the duration of the no-lick period. This iterative addition could be repeated for a maximum of five times. Therefore, the entire duration of the no-lick period could range between 1 and 25 s and depended on the animal’s ability to successfully suppress its licking.

Phase three is the two-armed bandit task. The trial timing is the same as phase 2, including the no-lick period before the go cue. Trials were organized in blocks with each block having a different set of reward probabilities. In a 70:10 block, the left lick spout carried a 70% likelihood of delivering water if chosen and the right lick port carried a 10% likelihood of delivering water if chosen. Conversely, in a 10:70 block, the reward probabilities for the left and right ports were 10% and 70%, respectively. At the beginning of each session, the block type (70:10 or 10:70) was randomly chosen. An uncued transition in block type occurred when the mouse chose the higher value side in 10 trials and then performed an additional random number of trials (L_Random_) with value drawn from a truncated geometric distribution (μ = 11, minimum = 0, maximum = 30). The session would terminate when the animal did not respond for 20 consecutive trials. Animals were deemed to be experts if they chose the side with higher reward probability for at least 50% of the trials over three consecutive sessions. We analyzed the data from sessions after animals reached expert performance.

### Statistics

All data are displayed as means ± SE or as box plots (medians, quartiles, and 90% tails) with individual points overlaid. Sample sizes were chosen based on standards in the field. No assumptions were made for data distribution, and the specific statistical tests used and relevant values are noted in the figure legends. Significant level was set for an α level of 0.05, and multiple comparisons corrections were used when appropriate. Statistical analyses were performed using the Real Statistic Pack plugin for Microsoft Excel (Release 8.0).

## Results

### *Scn2a*^+/−^ mice learn the foraging task similar to WT littermates

**Figure 1.**
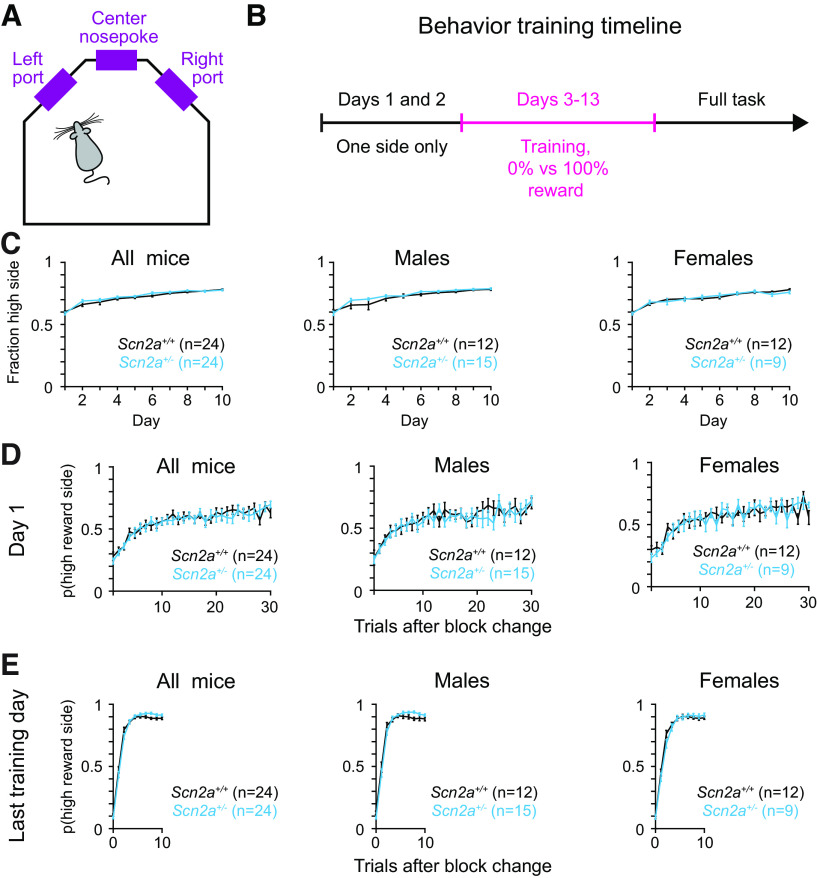
*Scn2a*^+/−^ mice learn the foraging task as well as WT littermates. ***A***, Schematic of the behavioral box. Animals self-initiate trials in the center nosepoke and subsequently choose between the left and right port for reward. ***B***, Timeline of behavioral training. On days 1 and 2, animals learn to receive reward from only the left or right port, 100 trials per day. Day 3 onward, animals learn to receive reward from both the left and right ports. Reward probabilities across ports are 0% and 100%, and the reward contingencies switch after 100 trials or after 9/10 correct choices. Animals train for 10 d or until they complete two sessions >80% correct, whichever comes first. After training, animals are put on the full behavioral task (details in Materials and Methods). Pink denotes the section used for analysis (in ***C–E***, day 1 corresponds to day 3 of the training timeline). ***C***, Probability of choosing the 100% baited port across training sessions across all animals (left), males (center), or females (right). *Scn2a*^+/−^ mice in cyan and WT littermates in black. Bars are means ± SEM. All animals: *p* = 0.382 for genotype and day; males: *p* = 0.379 for genotype and day; females: *p* = 0.451 for genotype and day, repeated measures two-way ANOVA. ***D***, Probability of choosing the new 100% baited port after the reward contingences were reversed on day 1 of the training block across all animals (left), males (center), and females (right). *Scn2a*^+/−^ mice in cyan and WT littermates in black. Bars are means ± SEM. All animals: *p* = 0.950 for genotype and trial; males: *p* = 0.950 for genotype and trial; females: *p* = 0.630 for genotype and trial, repeated measures two-way ANOVA. ***E***, Probability of choosing the new 100% baited port after the reward contingences were reversed on the final training day for each mouse across all animals (left), males (center), and females (right). Note that only 10 trials are plotted here as animals are well-trained and experience uncued block changes after fewer trials than on day 1. *Scn2a*^+/−^ mice in cyan and WT littermates in black. Bars are means ± SEM. All animals: *p* = 0.002 for genotype and trial; males: *p* = 0.014 for genotype and trial; females: *p* = 0.140 for genotype and trial, repeated measures two-way ANOVA.

To test behavioral flexibility and learning dynamics in conditions of *Scn2a* haploinsufficiency, we implemented a dynamic foraging task that has been shown to involve corticostriatal circuits and midbrain dopamine signaling (Samejima et al., 2005; Kennerley et al., 2006; Ragozzino, 2007; Lau and Glimcher, 2008; Rutledge et al., 2009; Hamid et al., 2016; Del Arco et al., 2017; Ueda et al., 2017; Donahue et al., 2018; Nakayama et al., 2018; Bari et al., 2019; Cox et al., 2023). Briefly, water deprived and freely moving mice learned to self-initiate trials in the center nosepoke and subsequently to choose between two reward ports with differing reward probabilities (Fig. 1*A*). Animals acquired the behavioral task over a 12d training period, the majority of which was spent on a version of the task where reward probabilities on the two ports were fixed at 0% and 100%. The location of the high reward probability port underwent uncued reversals after 100 trials or after the animal selected the high reward port 9 of the 10 previous trials (Fig. 1*B*). Animals performed this version of the task for 10 trials or until they reached 80% performance on two adjacent sessions (criterion).

We first asked whether *Scn2a*^+/−^ mice took longer to learn the behavior than their wild-type (WT) littermates. For each training day and animal, we calculated the fraction of trials where the animal chose the high reward port. *Scn2a*^+/−^ and WT mice did not differ in their learning curves, and there was no difference across sexes (Fig. 1*C*). Since both genotypes selected the high reward side equally across sessions, we next asked whether the two genotypes adapted behaviors differently after reward contingencies reversed. For each animal, we calculated the probability that the animal chose the high reward side on trials after a block change. *Scn2a*^+/−^ and WT mice did not show differences in their behavioral adaption on the first day of training (Fig. 1*D*).

Once animals were well-trained on the task (the day animals reached criterion or day 10), there was also no difference in *Scn2a*^+/−^ animals’ ability to reverse after a block reversal (Fig. 1*E*; note that animals perform the task very well once they reach criterion and often experienced block reversals after 10 trials). If anything, heterozygous mice tended to pick the higher reward side slightly more frequently later in the block (Fig. 1*E*). Overall, there was also no difference in performance across sex on days 1 and 10, although male *Scn2a*^+/−^ mice were slightly more likely to pick the higher reward size mid-block than their WT littermates (Fig. 1*D*,*E*). These data suggest that *Scn2a*^+/−^ mice do not have any deficits in their ability to learn the rules of a foraging task or to adapt their behavior when reward contingencies change.

### *Scn2a*^+/−^ mice perform the foraging task as well as WT littermates

**Figure 2.**
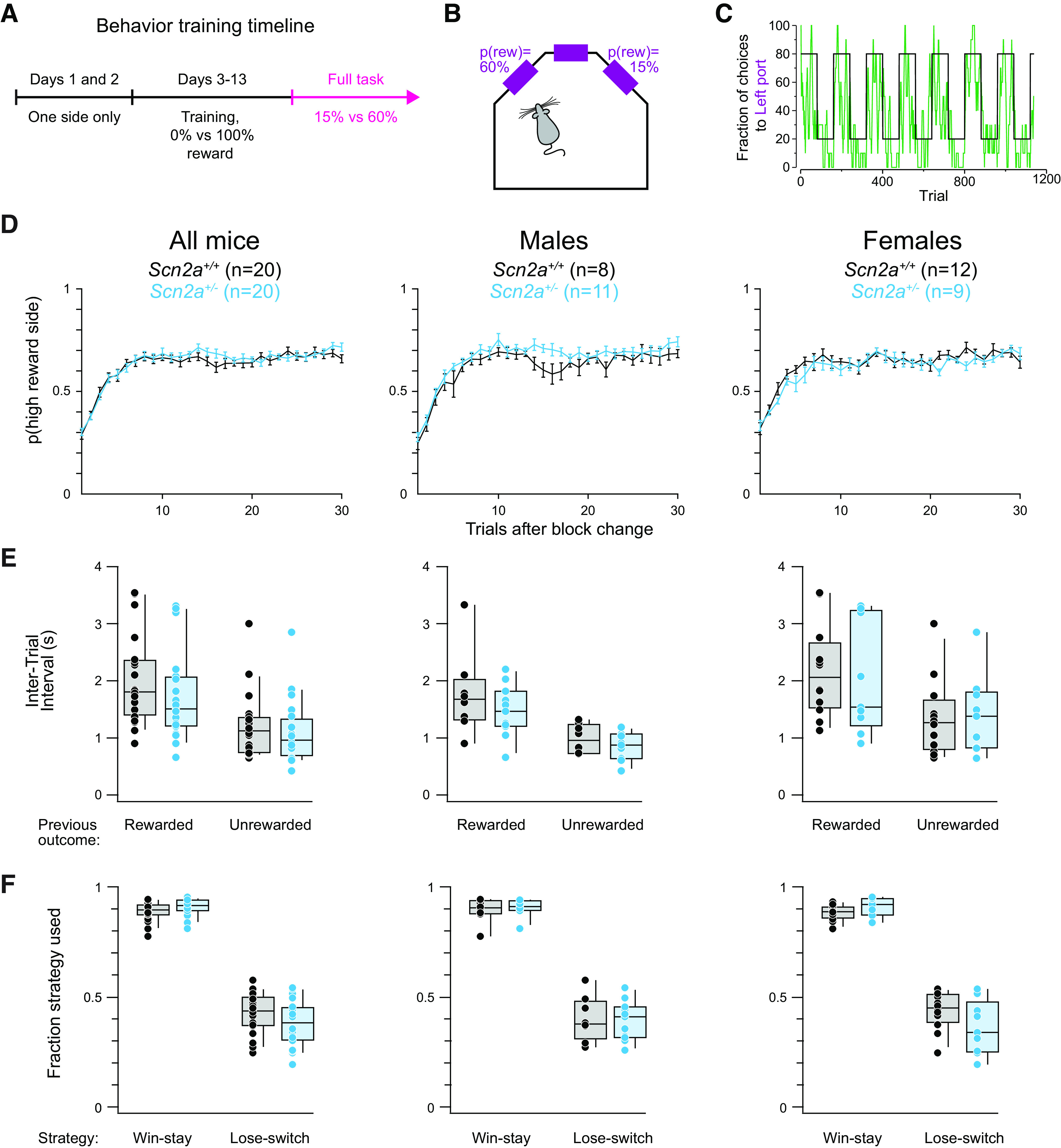
*Scn2a*^+/−^ mice perform the foraging task as well as WT littermates. ***A***, Timeline of the behavioral training. Pink denotes section used for analysis. ***B***, Schematic of the behavioral box. In this task, ports are set to 15% or 60% reward probabilities. ***C***, Choice behavior from an example animal (green, 10-trial moving average) in response to changes in reward contingencies [black, ratio of left to right side reward probabilities; e.g., 60/(15 + 60) or 15/(15 + 60)]. ***D***, Probability of choosing the new 60% baited port after the reward contingencies were reversed across all animals (left), males (center), and females (right). *Scn2a*^+/−^ mice in cyan and WT littermates in black. Bars are means ± SEM. All animals: *p* = 0.543 for genotype and trial; males: *p* = 0.046 for genotype and *p* = 0.682 for genotype and trial; females: *p* = 0.680 for genotype and trial, repeated measures two-way ANOVA. ***E***, Intertrial interval (ITI), measured as the time from when the animal left a side port to when it next entered the center poke, broken down by genotype and previous trial outcome for all animals (left), males (center), and females (right). Circles are individual animals. All WT, previously rewarded: median 1.8 s, interquartile range (IQR) 1.4–2.4 s; all *Scn2a*^+/−^, previously rewarded: median 1.5 s, IQR 1.2–2.1 s; all WT, previously unrewarded: median 1.1 s, IQR 0.7–1.4 s; all *Scn2a*^+/−^, previously unrewarded: median 1.0 s, IQR 0.7–1.3 s; male WT, previously rewarded: median 1.7 s, IQR 1.3–2.0 s; male *Scn2a*^+/−^, previously rewarded: median 1.5 s, IQR 1.2–1.8 s; male WT, previously unrewarded: median 1.0 s, IQR 0.7–1.2 s; male *Scn2a*^+/−^, previously unrewarded: median 0.9 s, IQR 0.6–1.1 s; female WT, previously rewarded: median 2.1 s, IQR 1.5–2.7 s; female *Scn2a*^+/−^, previously rewarded: median 1.5 s, IQR 1.2–3.2 s; female WT, previously unrewarded: median 1.3 s, IQR 0.8–1.7 s; female *Scn2a*^+/−^, previously unrewarded: median 1.4 s, IQR 0.8–1.4 s. Rewarded versus unrewarded, control: *p* < 0.001; rewarded versus unrewarded, *Scn2a*^+/−^: *p* = 0.002. Rewarded versus unrewarded, male controls: *p* = 0.005; rewarded versus unrewarded, *Scn2a*^+/−^ males: *p* = 0.001. Two-way ANOVAs followed by pairwise Mann–Whitney tests and Bonferroni correction. ***F***, The fraction of win-stay and lose-switch trials for all mice (left), males (center), and females (right). Circles are individual animals. All WT, win-stay: median 0.90, IQR 0.87–0.92; all *Scn2a*^+/−^, win-stay: median 0.92, IQR 0.89–0.94; all WT, lose-switch: median 0.44, IQR 0.37–0.50; all *Scn2a* lose-switch: median 0.38, IQR 0.30–0.45; male WT, win-stay: median 0.91, IQR 0.88–0.94; male *Scn2a*^+/−^, win-stay: median 0.91, IQR 0.89–0.94; male WT, lose-switch: median 0.38, IQR 0.31–0.48; male *Scn2a* lose-switch: median 0.41, IQR 0.31–0.45; female WT, win-stay: median 0.89, IQR 0.86–0.91; female *Scn2a*^+/−^, win-stay: median 0.92, IQR 0.87–0.95; female WT, lose-switch: median 0.45, IQR 0.38–0.41; female *Scn2a* lose-switch: median 0.34, IQR 0.25–0.48. No difference across genotypes, two-way ANOVAs followed by pairwise Mann–Whitney tests and Bonferroni correction. See Extended Data Figure 2-1 for behavioral data on a similar, head-fixed version of the task.

During training described above, reward contingencies were fixed at 0% and 100%. These reward contingencies taught the animals to sample both ports and to adapt their behavior across block changes, but did not encourage the animals to dynamically sample both reward ports within a given block. We therefore wanted to test the *Scn2a*^+/−^ mice on a version of the behavior where the chance of getting a reward is probabilistic on both sides for every trial and where animals would have to rely on their recent choice and outcome histories to guide future choices. To achieve this, we used the dynamic version of the behavior where the reward availability for each port was independently assigned to 60% for one port (high reward probability) and 15% for the other (low reward probability; Fig. 2*A–C*). Once a reward was assigned to a port, the reward was available until the animal chose that port (Sugrue et al., 2004; Lau and Glimcher, 2008; Fonseca et al., 2015; Bari et al., 2019). The location of the high reward probability port underwent uncued reversal to the opposite port every 80 trials. Animals were tested on the dynamic foraging task after reaching criterion in the training task (Figs. 1*B*, 2*A*). The fraction of choices the animals made to a side “matched” the reward probability for that side, similar to what was noted in other studies (Sugrue et al., 2004; Lau and Glimcher, 2008; Fonseca et al., 2015; Tsutsui et al., 2016; Bari et al., 2019; Fig. 2*C*).

10.1523/ENEURO.0367-23.2023.f2-1 Extended Data Figure 2-1 Performance of *Scn2a^+/-^* mice and wild-type littermates in a head-fixed version of the probabilistic reward task. ***A***, Schematic of the behavioral task. On each trial, a head-fixed mouse makes a choice via a left or right tongue lick following an auditory go cue. Depending on the reward probabilities, the choice may lead to a water reward. Trials are organized in blocks with each block having a different set of reward probabilities, including “70:10” (70% chance of reward on the left side; 10% on the right) and “10:70” (10% on the left; 70% on the right). Uncued block switches occur when the animal satisfies the switching condition, which is to choose the side with higher reward probability for 10 trials and then perform an additional random number of trials drawn from a truncated exponential distribution. ***B***, Performance in an example session for a *Scn2a^+/-^* mouse. Top, Reward probabilities. Bottom, Choice behavior and the outcome for each trial. Red bar, Left choice. Blue bar, Right choice. Black bar, Reward. ***C***, Similar to ***B*** for a wild-type littermate mouse. ***D***, Probability of choosing the side with high reward probability after a block transition for *Scn2a^+/-^* mice (blue) and wild-type littermates (black). Mean ± SEM. *N* = 3 *Scn2a^+/+^* mice (2 male, 1 female) and *N* = 3 *Scn2a^+/-^* mice (1 male, 2 female). ***E***, Fraction of trials in which mice employ win-stay or lose-switch strategies. Circle, Individual animals. Mean ± SEM. Download Figure 2-1, EPS file.

We first asked whether *Scn2a*^+/−^ mice were less flexible in adapting their behavior after a block change under this more unpredictable reward paradigm relative to WT littermates. However, both *Scn2a*^+/−^ mice and WT littermates flexibly changed their choice behavior after a block change and began preferring the higher rewarded port (Fig. 2*D*). Across the overall population, there was no difference across genotype, although in males, there was a statistically significant difference across genotype largely driven by mid-block performance (Fig. 2*D*).

Since there appeared to be no difference in behavioral flexibility across genotypes, we next asked whether there might be a difference in the intertrial interval (ITI), as this metric can serve as a proxy for motivational state (A.Y. Wang et al., 2013; Hamid et al., 2016; Cox et al., 2023). Because individual trials are self-initiated, we defined ITI to be the time from which the animal exited a side port to when it next entered the center nosepoke. We analyzed trials in which the animal previously received a reward and trials in which it did not separately, as we hypothesized that there may be a difference in motivational state based on previous reward as shown in other studies (Cox et al., 2023). In both the overall population and among males, WT and *Scn2a*^+/−^ mice took longer to initiate a new trial after receiving a reward, but there was no ITI difference between *Scn2a*^+/−^ mice and WT littermates in either previously rewarded or unrewarded conditions or across sexes (Fig. 2*E*).

Finally, we assessed whether *Scn2a*^+/−^ mice and WT littermates might be using different strategies to optimize reward. One possible strategy is win-stay, lose-switch, in which animals repeat their choice if they just received a reward or pick the opposite choice if they did not (Evenden and Robbins, 1984; Sugrue et al., 2004; Lau and Glimcher, 2008; Donahue et al., 2018). We analyzed the fraction of trials in which *Scn2a*^+/−^ mice and WT littermates chose the same port after receiving a reward (“win-stay”) or changed their choice after receiving no reward (“lose-switch”). Both *Scn2a*^+/−^ mice and WT littermates tended to revisit the same port after receiving reward and switched ports <50% of the time after a “lose” trial, but there was no difference across genotypes or sex (Fig. 2*F*).

In a separate set of experiments, we tested an independent sample of *Scn2a*^+/−^ mice and WT littermates on a similar, head-fixed version of this task (Extended Data Fig. 2-1*A*). Animals of both genotypes acquired and performed the task (Extended Data Fig. 2-1*B*,*C*). As with the freely moving version of the behavior, there was no difference across genotype in successfully switching sides after a block reversal (Extended Data Fig. 2-1*D*) or in win-stay, lose-switch strategies (Extended Data Fig. 2-1*E*). Of note, the head-fixed assays were done in a different lab than those with freely-moving animals. Taken together, the freely-moving and head-fixed behavioral results suggest that *Scn2a*^+/−^ mice and WT littermates perform comparably and use similar strategies in this dynamic foraging task, although the head-fixed results should be considered preliminary given the lower number of animals assayed herein.

### Varying block length did not affect *Scn2a*^+/−^ mouse performance

**Figure 3.**
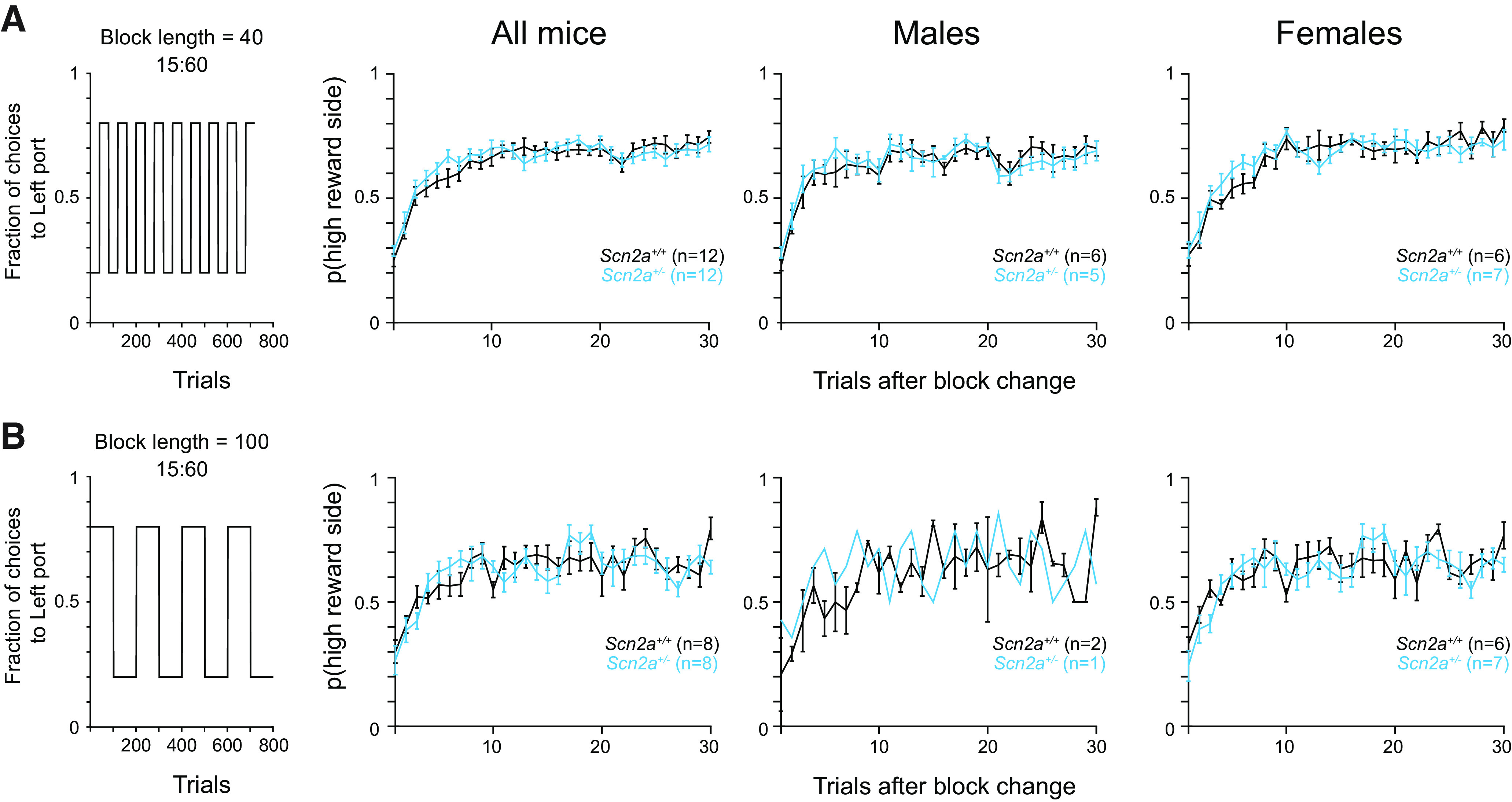
Varying block length did not affect *Scn2a*^+/−^ mouse performance. ***A***, Left, Plot of reward contingencies for a session with a block length of 40 and 15%/60% reward probabilities. Right, Probability of choosing the new 60% baited port after the reward contingencies were reversed across all animals, males, and females. Bars are means ± SEM. All animals: *p* = 0.644 for genotype and trial; males: *p* = 0.997 for genotype and trial; females: *p* = 0.638 for genotype and trial, repeated measures two-way ANOVA. ***B***, Left, Plot of reward contingencies for a session with a block length of 100 and 15%/60% reward probabilities. Right, Probability of choosing the new 60% baited port after the reward contingencies were reversed across all animals, males, and females. *Scn2a*^+/−^ mice in cyan and WT littermates in black. Bars are means ± SEM. All animals: *p* = 0.157 for genotype and trial; males: *p* = 0.573 for genotype and trial; females: *p* = 0.213 for genotype and trial, repeated measures two-way ANOVA.

We initially tested a block length of 80 trials for the dynamic foraging task, as this was long enough to provide sufficient trials for mice to learn new reward contingencies while still ensuring that animals can adapt their behavior without developing side biases or alternate strategies. However, we wondered whether changing the block length might unmask a behavioral difference across genotypes. Therefore, we ran a subset of mice on a version of the dynamic foraging task using a block length of 40 (Fig. 3*A*) or 100 trials (Fig. 3*B*). No differences between *Scn2a*^+/−^ and WT mice were observed across any behavior, though we note that the male cohort was under-powered for the 100-block experiment.

### Varying reward contingencies did not affect *Scn2a*^+/−^ mouse performance

**Figure 4.**
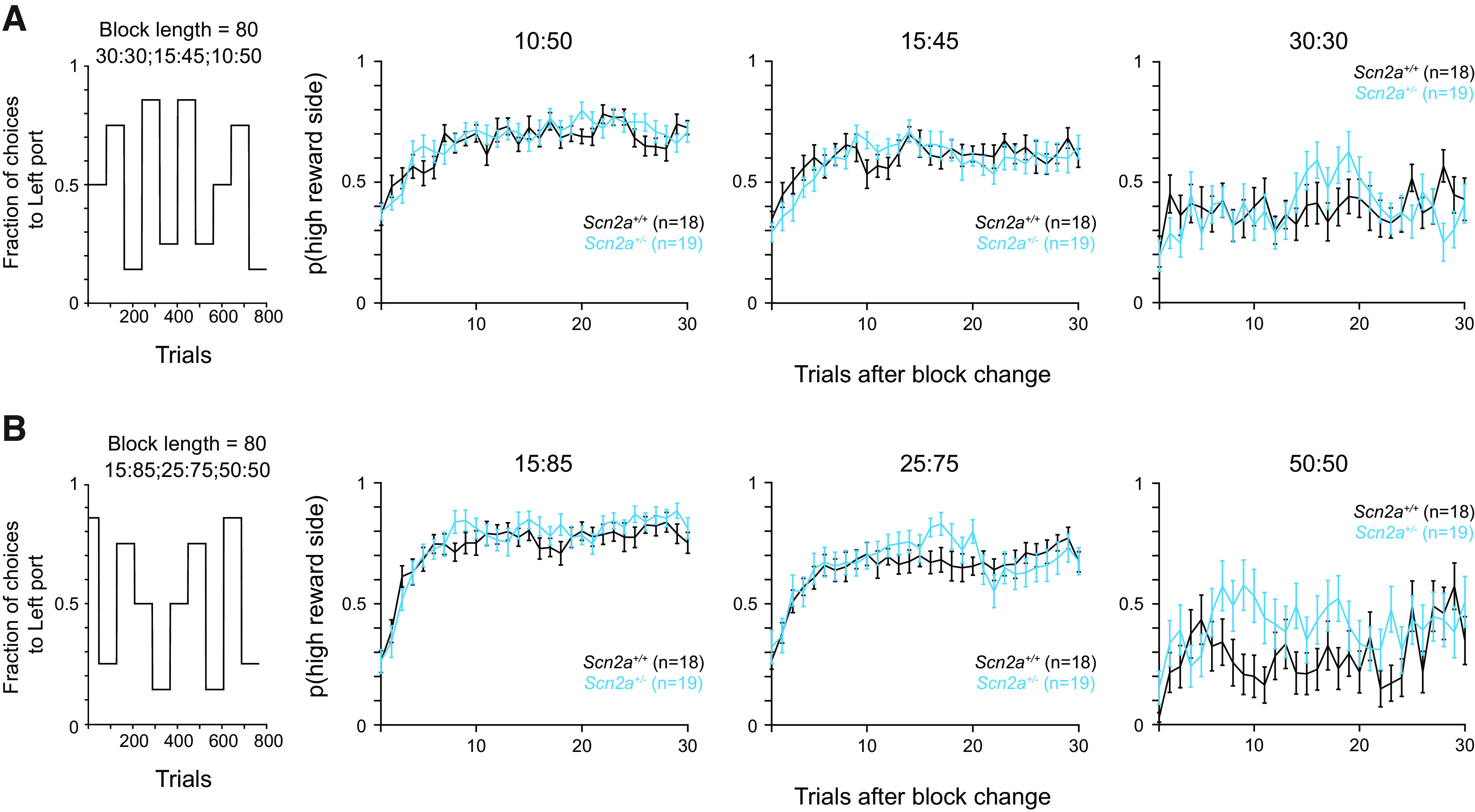
Varying reward contingencies did not affect *Scn2a*^+/−^ mouse performance. ***A***, Left, Plot of reward contingencies for a session with a block length of 80 and 30% versus 30%, 15% versus 45%, and 10% versus 50% reward probabilities. Right, Probability of choosing the high reward port (when applicable) for the 10% versus 50%, 15% versus 45%, and 30% versus 30% contingencies across all animals. *Scn2a*^+/−^ mice in cyan and WT littermates in black. Bars are means ± SEM 10% versus 50%: *p* = 0.351 for genotype and trial; 15% versus 45%: *p* = 0.689 for genotype and trial; 30% versus 30%: *p* = 0.150 for genotype and trial, repeated measures two-way ANOVA. ***B***, Left, Plot of reward contingencies for a session with a block length of 80 and 15% versus 85%, 25 versus 75%, and 50% versus 50% reward probabilities. Right, Probability of choosing the high reward port (when applicable) for the 15% versus 85%, 25% versus 75%, and 50% versus 50% contingencies across all animals. Bars are means ± SEM 15% versus 85%: *p* = 0.843 for genotype and trial; 25% versus 75%: *p* = 0.669 for genotype and trial; 50% versus 50%: *p* = 0.318 for genotype and trial, repeated measures two-way ANOVA.

*Scn2a*^+/−^ mice and WT littermates did not appear to perform differently in the dynamic foraging task with block lengths of 40, 80, and 100 trials and reward contingencies of 15% and 60%. As a final experiment, we tested whether varying the reward contingencies within a given experiment might make it more difficult for mice to flexibly adapt their choice behavior. In a subset of behavioral sessions, we tested mice on a version of the dynamic foraging task where reward contingencies varied between 30%:30%, 15%:45%, and 10%:50% in each block of 80 trials (Fig. 4*A*). In another subset of sessions, we tested the same groups of mice on a version of the task where reward contingencies varied between 50%:50%, 15%:85%, and 25%:75% (Fig. 4*B*). We chose these reward contingencies as some were easily distinguishable (e.g., 15%:85%), some forced chance performance (e.g., 50%:50%), and some fell into an intermediate zone that is more difficult to discern (e.g., 15%:45%). Across these different reward contingencies, *Scn2a*^+/−^ mice and WT littermates were both able to adapt to the different reward contingencies (Fig. 4*A*,*B*). We did not test 0%:100% reward here, since well-trained animals did not show a behavioral difference with this reward contingency during training (Fig. 1*E*). Overall, our data suggest that *Scn2a*^+/−^ haploinsufficiency does not affect animals’ performance on a cognitively challenging dynamic decision-making behavioral task.

### *Scn2a*^+/−^ mice with seizures did not perform differently on behavioral tasks

In humans, *SCN2A* loss-of-function (LoF) is most commonly associated with ASD/ID. An estimated 20–30% of children with *SCN2A* LoF experience seizures, usually with an onset after the first postnatal year (Sanders et al., 2018; Brunklaus et al., 2022). While other groups have reported spontaneous “absence-like” seizures in *Scn2a*^+/−^ animals (Ogiwara et al., 2018), this does not phenocopy what is observed in human patients, and we have not observed spontaneous seizure-related phenotypes in our normally housed cohort of *Scn2a*^+/−^ mice of a similar age as those studied here (Tamura et al., 2022). Here, we observed spontaneous behavioral seizures associated with this study in four of 25 *Scn2a*^+/−^ mice (from several litters and breeding pairs, three males and one female, noted in different months; none observed in WT littermates). Of note, animals studied here were housed differently than the rest of our colony, with water consumption restricted in the home cage to increase motivation for 10% sucrose solution reward during behavior. All seizures were observed at the end of a day’s training when animals were being returned to their home cage. The four mice that exhibited seizures did not consume a different level of sucrose compared with animals that did not experience seizures (1.7 ± 0.12 ml of solution for four mice with seizure; 1.5 ± 0.14 ml for 11 *Scn2a*^+/−^ mice without seizure tested on same days, *p* = 0.2 Mann–Whitney *U* test). For one male animal, the seizure progressed from spontaneous convulsions to tonic clonus with hindlimb extension, followed by death. This occurred during the initial training, and the animal was therefore excluded from all analyses. For the three other animals (two male, one female), seizures were noted on two separate days each. All three animals exhibited spontaneous convulsions, “popcorning” behavior, with characteristic jumping within the cage, and myoclonic jerks with loss of balance. The two males also had seizures that progressed to tonic clonus with hindlimb extension. All three mice were tested on the entire behavior, and we did not exclude them from our analyses. We looked to see, however, whether seizures might have affected the behavioral performance of these three *Scn2a*^+/−^ mice.

**Figure 5.**
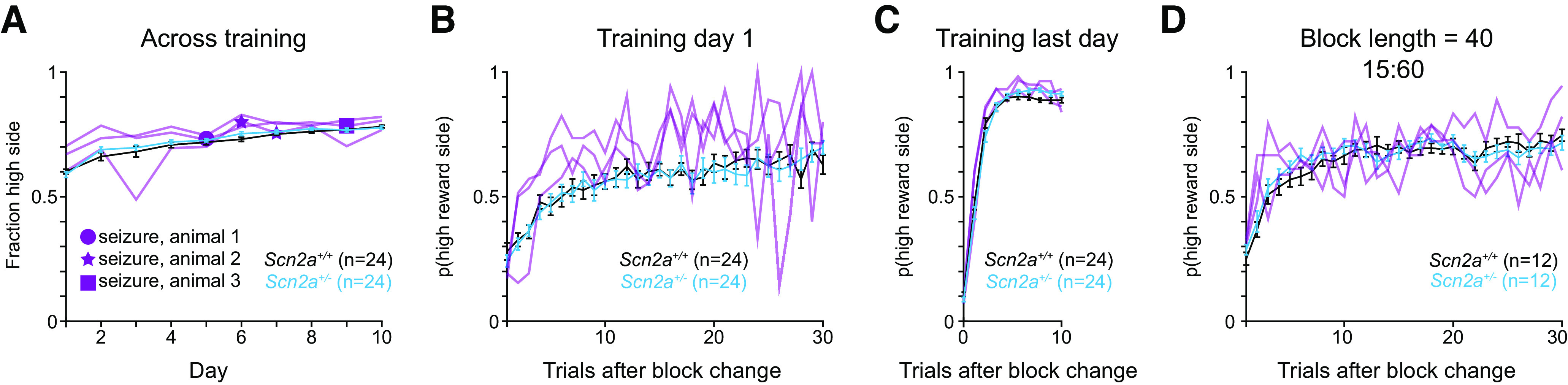
*Scn2a*^+/−^ mice with seizures did not perform differently on behavioral tasks. ***A***, Same as Figure 1*C*. Probability of choosing the 100% baited port across training sessions across all animals. *N* = 3 *Scn2a*^+/−^ mice (2 males, 1 female) with observed home cage seizures overlaid in purple (animals are included in *N* = 24 mean data). The days where individual animals experienced seizures are denoted with symbols. ***B***, Same as Figure 1*D*. Probability of choosing the new 100% baited port after the reward contingences were reversed on day 1 of the training block across all animals. *N* = 3 *Scn2a*^+/−^ mice with observed home cage seizures overlaid in purple (animals are included in *N* = 24 mean data). ***C***, Same as Figure 1*E*. Probability of choosing the new 100% baited port after the reward contingences were reversed on the final training day for each mouse across all animals. *N* = 3 *Scn2a*^+/−^ mice with observed home cage seizures overlaid in purple (animals are included in *N* = 24 mean data). ***D***, Same as Figure 3*A*. Probability of choosing the new 60% baited port after the reward contingencies were reversed (block length of 40 trials) across all animals. *N* = 3 *Scn2a*^+/−^ mice with observed home cage seizures overlaid in purple (animals are included in *N* = 12 mean data).

Their behavioral performance, measured by how quickly they adapted their behavior after a block reversal, was no different from other animals on either day 1 or the last day of training (Fig. 5). These animals were from two cohorts and thus were tested on different variations of the dynamic foraging task. They did not behave differently than other mice in any variation. As an example, all three were tested on the 40-trial block version of the dynamic foraging task (Fig. 3*A*) but showed no obvious behavioral differences (Fig. 5*D*). These data suggest that under certain conditions, seizures can be observed in *Scn2a* haploinsufficient animals but that they do not affect learning or performance of this foraging task.

## Discussion

The voltage-gated sodium channel Na_v_1.2 is expressed in medial prefrontal cortex (mPFC) as well as striatum and the axons of midbrain dopamine neurons (Miyazaki et al., 2014; Spratt et al., 2019; Yang et al., 2019; Zhang et al., 2021). Previous work has demonstrated that corticostriatal activity and dopaminergic signaling contribute to choice behaviors including in dynamic foraging tasks (Hamid et al., 2016; Bari et al., 2019; Cox et al., 2023). We therefore hypothesized that *Scn2a* haploinsufficiency may affect how animals learn or perform a decision-making behavior. Here, we show that *Scn2a*^+/−^ mice exhibit no differences in learning and performing a dynamic foraging behavior, even under increased task demands (varied reward contingencies and block lengths).

### Reduced *Scn2a* expression across relevant brain regions did not translate into a behavioral phenotype

Since dysfunction in *SCN2A* is highly associated with autism spectrum disorder and intellectual disability (ASD/ID; Sanders et al., 2012; Ben-Shalom et al., 2017; Satterstrom et al., 2020; Fu et al., 2022), substantial effort has been put toward understanding the cellular and behavioral changes linked to *Scn2a* haploinsufficiency. Given that children with *SCN2A* loss-of-function (LoF) variants have notable behavioral impairments (Sanders et al., 2018) and given Na_v_1.2’s contribution to dendritic and axonal excitability (Bender and Trussell, 2012; Kole and Stuart, 2012; Spratt et al., 2019), it is somewhat surprising that more overt learning deficits have not been observed in *Scn2a* heterozygous mice. In one study, *Scn2a*^+/−^ mice took longer to learn an H-maze task (Middleton et al., 2018). Previously, it was shown that found that male, but not female, *Scn2a*^+/−^ mice were a little slower to learn the reversed contingencies in a water T-maze (Spratt et al., 2019). *Scn2a*^+/−^ mice were ultimately able to reach wild-type (WT)-level performance in both tasks. This is consistent with our findings that observed neither a learning nor performance difference. Other studies have found that *Scn2a* haploinsufficiency resulted in modest differences in various behavioral assays, including the open field, elevated plus maze, resident-intruder task, and fear conditioning (Shin et al., 2019; Tatsukawa et al., 2019). However, these effects were not replicated consistently across studies (Shin et al., 2019; Spratt et al., 2019; Zhang et al., 2021).

Given this, it remains a challenge to connect robust cellular deficits observed in cases of *Scn2a* loss to behavioral readouts in heterozygotes that can be observed reliably across research groups. These difficulties are mirrored by similar issues across multiple mouse models of ASD-associated genes, especially when studied in differing strains (Silverman et al., 2022; Tabbaa et al., 2023). For studies of *Scn2a*, one potential solution would be to leverage conditional knock-out or gene-trap approaches to reduce *Scn2a* expression by >50% in circuits of interest (Eaton et al., 2021; Spratt et al., 2021). In gene-trap *Scn2a*-deficient mouse models, where *Scn2a* expression is ∼25% that of WT animals, overt alterations in several behaviors and sleep dynamics were observed (Eaton et al., 2021; Ma et al., 2022).

Beyond *Scn2a* dosage, one could also focus more on more reflexive behaviors in mouse, as circuitry underlying such behaviors is often highly conserved across species. For example, our lab found recently that oculomotor reflexes are altered robustly by *Scn2a* haploinsufficiency (C. Wang et al., 2023). These reflexes are controlled in part by cerebellar circuits that are evolutionarily ancient and present in all vertebrates. Thus, conserved function of Na_V_1.2 in cerebellar circuits and their involvement in behaviors that are not routinely considered in studies of ASD models (Silverman et al., 2010) may nevertheless offer high face validity across species.

Lastly, there is a final consideration of how mouse behavior relates to human behavior, and whether face validity in core ASD/ID-related behaviors (e.g., social interaction, communication, learning) could be captured appropriately in mouse models (Silverman et al., 2022). Towards this end, other animal models may be more appropriate for different traits. For example, rats can learn and perform more complex behavioral tasks than mice, potentially allowing for study of aspects of learning more closely related to those engaged by children (Wong et al., 2020; Fernandes et al., 2021; Harris et al., 2021; Anstey et al., 2022). Furthermore, other rodent species, like prairie voles, form highly complex social networks and enduring relationships that persist throughout life, allowing for study of social interaction and attachment phenotypes that are not innate to mouse (Young et al., 2002; McGraw and Young, 2010). Lastly, nonhuman primate models heterozygous for ASD-associated genes can better recapitulate ASD-associated behaviors in conditions where mouse models fail (Jiang and Ehlers, 2013; Jennings et al., 2016; Zhou et al., 2019). Overall, this suggests that, depending on the behavior in question, leveraging different animal models may be warranted.

### Potential effect of housing conditions on seizure susceptibility

An estimated 20–30% of children with loss-of-function *SCN2A* variants develop epilepsy, often after the first year of life (Sanders et al., 2018). Excess spike-and-wave discharge activity has been noted in some, but not all recordings from *Scn2a*^+/−^ mice (Ogiwara et al., 2018; Tamura et al., 2022). To our knowledge, observations here are the first of spontaneous, “popcorning” behavioral seizures in *Scn2a*^+/−^ animals, with 16% of our *Scn2a*^+/−^ cohort exhibiting behavioral seizures. This suggests that conditions used to motivate behavior, including water restriction and sucrose reward consumption, may increase seizure susceptibility in *Scn2a* haploinsufficient conditions. The seizures that we observed all occurred after the animals were returned to their homecage after a behavioral training session with sucrose reward, and we speculate that changes in hydration or glucose levels could contribute to seizure susceptibility, as has been reported previously (Gibbs, 1939; Andrew, 1991; Schwechter et al., 2003; Reid et al., 2011). Increasingly, there is an appreciation for potential metabolic alterations underlying epilepsies in channelopathies (Neal et al., 2023). These data suggest that *Scn2a*^+/−^ mice are more susceptible to seizures related to fluid/energy homeostasis. Future studies could focus on external stressors such as fever or fasting to better understand mechanisms that may result in seizures in *Scn2a* haploinsufficiency. Importantly, however, the three animals that survived multiple seizures successfully learned and performed the behavioral task (Fig. 5). These observations suggest that water restriction and/or increased sucrose consumption may increase the likelihood of seizures in these animals, but also that, even with seizures, *Scn2a*^+/−^ mice can learn the dynamic foraging behavior and adapt their choice behavior.

## Synthesis

Reviewing Editor: David Schoppik, New York University Decisions are customarily a result of the Reviewing Editor and the peer reviewers coming together and discussing their recommendations until a consensus is reached. When revisions are invited, a fact-based synthesis statement explaining their decision and outlining what is needed to prepare a revision will be listed below. The following reviewer(s) agreed to reveal their identity: Sophie Hill.

Major Comments:

1. The motivation for this study, as detailed in the manuscript, is profoundly under-developed. Consequentially it wasn’t clear whether this paper should be published at all. The reviewers struggled to understand why, given the literature (364-382), someone would expect a different outcome. The argument in 381-382 that the results “aren’t consistently replicated across studies” is much too vague to be helpful. Part of the challenge here is eNeuro’s double-blind process: under other circumstances the Introduction/Discussion could refer to prior work with these mice, here that isn’t possible. That said, we all saw this as an opportunity. We encourage the authors to make the case for doing the initial experiments sufficiently strong. Surely there were good reasons behind doing the initial work. The manuscript would be considerably stronger for justifying the effort without reference to the authors’ past experiences/expertise.

 2. In the results, the description of the head-fixed experiment is very limited. It’s unclear why the authors undertook it (it’s also not mentioned in the abstract, introduction, or discussion). More explanation is needed. Finally, the “smaller cohort” with an n=3 is simply too low to draw any conclusions.

3. To Fig. 5, the authors could add some indication of when the seizures took place for each mouse (perhaps in Fig. 5A).

Minor points:

1. Abstract - minor quibble, but the phrase “where loss of one copy of a gene cannot be compensated for by its partner” is a bit unscientific.

2. 3. Description of Scn2a expression patterns (lines 40-48): The statement that “Scn2a is expressed predominantly in pyramidal neurons” is not supported by the literature. Most investigations of gene expression show only small differences in sodium channel gene expression between neuronal cell types, and Scn2a is expressed in inhibitory neurons. It’s also confusing that the authors go on to say that “Scn2a is also expressed widely in the brain..., including striatal projection neurons ... and midbrain dopamine neurons”.

4. In lines 55-56, the authors state “Several mouse models of ASD have recapitulated aspects of this phenotype across different behavioral paradigms including the Morris water maze and Y-maze”. How consistent is this finding among different ASD models? Have similar dynamic foraging tasks been conducted in other ASD models?

5. The phrasing in line 323 is ambiguous. Are the authors saying that Ogiwara et al observed spontaneous, subclinical (I assume this means non-behavioral) seizures? The ambiguous phrasing is repeated in line 418. More detail on the EEG abnormalities observed could clarify this point.

6. Line 386 - do the authors mean a mixed genetic background (e.g. B6 X C3H) or mice heterozygous for Scn2a mutations?

7. The methods refer to t-tests, but ANOVA seems to have been used in many cases. Please clarify.

## Author Response

We thank the editor and reviewers for their comments and suggestions for our manuscript. This is our first experience with a consensus review at eNeuro, and find it to be a very positive approach-if only more journals went this route!

To address these reviews, we expanded the text to provide better motivation of the overall question and goals of the study, including the head-fixed portion. We have also clarified the text where appropriate and edited Figure 5A to highlight when animals had seizures. These suggestions most definitely strengthened the manuscript, and we thank the reviewers for their thoughtfulness in providing these comments.

Below are unredacted reviewer comments in black and our responses in blue.

Sincerely,

Selin Schamiloglu

Alex Kwan

Kevin Bender

1. The motivation for this study, as detailed in the manuscript, is profoundly under-developed. Consequentially it wasn’t clear whether this paper should be published at all. The reviewers struggled to understand why, given the literature (364- 382), someone would expect a different outcome. The argument in 381-382 that the results “aren’t consistently replicated across studies” is much too vague to be helpful. Part of the challenge here is eNeuro’s double-blind process: under other circumstances the Introduction/Discussion could refer to prior work with these mice, here that isn’t possible. That said, we all saw this as an opportunity. We encourage the authors to *make the case for why the initial experiments were undertaken as strongly as possible.* Surely there were good reasons behind doing the initial work. The manuscript will benefit from for justifying the effort without reference to the authors’ past experiences/expertise.

We thank the editor and reviewers for this suggestion, and have revised the introduction considerably. As you note, there is ample reason to have attempted this behavior in light of prior studies that noted little to no behavioral differences in Scn2a+/- animals.

Dynamic foraging requires the animals to use prior outcomes to guide current decisions. The task offers a unique opportunity to study reward-guided learning because it has several features that are different from assays used in prior studies. One, dynamic foraging requires learning in an uncertain environment. Choices are not associated with specific outcomes, but rather rewards are delivered probabilistically and the reward probabilities can switch over time, therefore the animals have to continually incorporate feedback from outcome (Soltani and Izquierdo, 2019). Two, dynamic foraging can be analyzed using computational models. Different models including reinforcement learning algorithms can be fitted to the animal’s choice data to yield insights into the decision-making process (Beron et al., 2022; Ito and Doya, 2015; Le et al., 2023). Three, dynamic foraging is translational. Because the task is non-trivial and challenging, it can be tested on non-human primates (Donahue and Lee, 2015) and humans (Sutton and Barto, 2018). Finally, four, numerous studies have shed light onto the neural circuits involved in dynamic foraging, implicating regions including medial frontal cortex (Atilgan et al., 2022), orbitofrontal cortex (Groman et al., 2019), retrosplenial cortex (Hattori et al., 2019), and the direct and indirect pathways in dorsal striatum (Tai et al., 2012). Relatedly, performance in dynamic foraging is impaired for dysfunctions such as rodent addiction models (Groman et al., 2018). For these reasons, we believe it was worthwhile to determine if Scn2a+/- mice, although exhibiting mild behavioral changes in other learning paradigms, may have specific deficits in the dynamic foraging task. If true, this could have set the stage for further computational modeling, translational comparisons, and circuit dissection studies. Alas, the current study did not reveal significant behavioral changes in dynamic foraging in mice with Scn2a haploinsufficiency.

2. In the results, the description of the head-fixed experiment is very limited. It’s unclear why the authors undertook it (it’s also not mentioned in the abstract, introduction, or discussion). More explanation is needed. Finally, the “smaller cohort” with an n=3 is simply too low to draw any conclusions.

This was tricky to describe due to the double-blind aspects of the review process and are writing the following to Dr. Schoppik in his role as editor. If this should be relayed to reviewers, we give you license to redact text as necessary.

The freely behaving work was all done in the Bender lab by Dr. Schamiloglu, and it was only through a chance encounter that we learned that Ms. Wu had done similar studies on head fixed mice in the Kwan lab. We decided to collaborate on this publication after comparing our work, and have revised the text to note that this was “an independent” group of mice. We feel that the addition of the six animals from the Kwan lab are an important addition to the manuscript for several reasons: One, they replicate what we observed using a similar behavior in head-fixed conditions. Two, that we independently performed the same experiment provides some extra motivation to publish this “negative” result. If we both chose to invest time in this approach, it is likely that others have thought along the same lines; publishing this could save others the effort.

We do appreciate that the number of head-fixed animals is low, but we highlight that animals performed a high number of sessions (21 sessions/246 blocks for WT littermates and 16 sessions/237 blocks for Scn2a+/- mice). Altogether, we felt that this work, despite the limited number of animals, was a net positive for the overall manuscript as it was an independent replication of this bandit task in a head-fixed condition. We now note in the results that this head-fixed “result” should be considered preliminary given the number of animals assayed.

Regarding commentary in the abstract and introduction: given the consistent negative results from both versions of the task, we did not distinguish between freely-moving or head-fixed in the abstract, introduction, or discussion.

3. To Fig. 5, the authors could add some indication of when the seizures took place for each mouse (perhaps in Fig. 5A).

We have modified Figure 5A to highlight when animals had seizures at the end their day’s training. Each seizure is noted with a different symbol per animal. There does not appear to be any overt different in performance on those days.

Minor points:

1. Abstract - minor quibble, but the phrase “where loss of one copy of a gene cannot be compensated for by its partner” is a bit unscientific.

We replaced “by its partner” with “by the other allele.”

2. 3. Description of Scn2a expression patterns (lines 40-48): The statement that “Scn2a is expressed predominantly in pyramidal neurons” is not supported by the literature. Most investigations of gene expression show only small differences in sodium channel gene expression between neuronal cell types, and Scn2a is expressed in inhibitory neurons. It’s also confusing that the authors go on to say that “Scn2a is also expressed widely in the brain..., including striatal projection neurons ... and midbrain dopamine neurons”.

Apologies for the lack of clarity here. That statement was intended to describe expression in neocortex. There, Scn2a mRNA is expressed in a range of cell classes, including interneurons, but this mRNA is not a good predictor of membrane expression in those interneurons. We and others have tested this specifically by examining AP waveform properties in PV+ and SOM+ cells in Scn2a+/- animals, as well as by examining immunofluorescence (Hu et al., 2009; Spratt et al., 2019a). To improve clarity, we have revised the associated sentence as such:

“In neocortex, Scn2a is expressed predominantly on the plasma membranes of pyramidal cells (Hu et al., 2009; Li et al., 2014; Yamagata et al., 2017), and previous work has shown that Scn2a haploinsufficiency in mice results in reduced dendritic excitability in prefrontal cortical pyramidal cells (Spratt et al., 2019b; Tamura et al., 2022; Nelson et al., 2022).”

Outside the neocortex, functional expression does include many other cell classes, and we have revised the associated paragraph to make this more clear as well.

4. In lines 55-56, the authors state “Several mouse models of ASD have recapitulated aspects of this phenotype across different behavioral paradigms including the Morris water maze and Y-maze”. How consistent is this finding among different ASD models? Have similar dynamic foraging tasks been conducted in other ASD models?

We expanded the introduction to discuss these behaviors in more detail.

5. The phrasing in line 323 is ambiguous. Are the authors saying that Ogiwara et al observed spontaneous, subclinical (I assume this means non-behavioral) seizures? The ambiguous phrasing is repeated in line 418. More detail on the EEG abnormalities observed could clarify this point.

Correct, Ogiwara described spike-and-wave discharges but did not relate these to behavior. We observed a popcorning-like seizure that has been linked to ictal spikes in other studies. We have clarified these two observations in text, noting their observations related to EEG whereas ours related only to behavior.

6. Line 386 - do the authors mean a mixed genetic background (e.g. B6 X C3H) or mice heterozygous for Scn2a mutations?

Sorry, we meant different mouse strains, and have adjusted the language to reflect this.

7. The methods refer to t-tests, but ANOVA seems to have been used in many cases. Please clarify.

For clarity, we included information on statistics in figure legends, and refer to the figure legends for statistics in the methods.

References

Atilgan H, Murphy CE, Wang H, Ortega HK, Pinto L, Kwan AC (2022) Change point estimation by the mouse medial frontal cortex during probabilistic reward learning.

Beron CC, Neufeld SQ, Linderman SW, Sabatini BL (2022) Mice exhibit stochastic and efficient action switching during probabilistic decision making. Proc Natl Acad Sci U S A 119:e2113961119.

Donahue CH, Lee D (2015) Dynamic routing of task-relevant signals for decision making in dorsolateral prefrontal cortex. Nat Neurosci 18:295-301.

Groman SM, Keistler C, Keip AJ, Hammarlund E, DiLeone RJ, Pittenger C, Lee D, Taylor JR (2019) Orbitofrontal Circuits Control Multiple Reinforcement-Learning Processes. Neuron 103:734-746.e3.

Groman SM, Rich KM, Smith NJ, Lee D, Taylor JR (2018) Chronic Exposure to Methamphetamine Disrupts Reinforcement-Based Decision Making in Rats. Neuropsychopharmacol 43:770-780.

Hattori R, Danskin B, Babic Z, Mlynaryk N, Komiyama T (2019) Area-Specificity and Plasticity of History-Dependent Value Coding During Learning. Cell 177:1858-1872.e15.

Hu W, Tian C, Li T, Yang M, Hou H, Shu Y (2009) Distinct contributions of Nav1.6 and Nav1.2 in action potential initiation and backpropagation. Nat Neurosci 12:996-1002.

Ito M, Doya K (2015) Parallel Representation of Value-Based and Finite State-Based Strategies in the Ventral and Dorsal Striatum. PLOS Computational Biology 11:e1004540.

Le NM, Yildirim M, Wang Y, Sugihara H, Jazayeri M, Sur M (2023) Mixtures of strategies underlie rodent behavior during reversal learning. PLOS Computational Biology 19:e1011430.

Li T, Tian C, Scalmani P, Frassoni C, Mantegazza M, Wang Y, Yang M, Wu S, Shu Y (2014) Action Potential Initiation in Neocortical Inhibitory Interneurons. PLOS Biology 12:e1001944.

Nelson AD, Catalfio AM, Gupta JM, Min L, Caballero-Floran RN, Dean KP, Elvira CC, Derderian KD, Kyoung H, Sahagun A, Sanders SJ, Bender KJ, Jenkins PM (2022) Physical and functional convergence of the autism risk genes Scn2a and Ank2 in neocortical pyramidal cell dendrites (preprint). Neuroscience.

Soltani A, Izquierdo A (2019) Adaptive learning under expected and unexpected uncertainty. Nat Rev Neurosci 20:635-644.

Spratt PWE, Ben-Shalom R, Keeshen CM, Burke KJ, Clarkson RL, Sanders SJ, Bender KJ (2019) The Autism-Associated Gene Scn2a Contributes to Dendritic Excitability and Synaptic Function in the Prefrontal Cortex. Neuron 103:673-685.e5.

Sutton RS, Barto AG (2018) Reinforcement Learning, second edition: An Introduction. MIT Press.

Tai L-H, Lee AM, Benavidez N, Bonci A, Wilbrecht L (2012) Transient stimulation of distinct subpopulations of striatal neurons mimics changes in action value. Nat Neurosci 15:1281-1289.

Tamura S et al. (2022) CRISPR activation rescues abnormalities in SCN2A haploinsufficiency-associated autism spectrum disorder (preprint). Neuroscience.

Yamagata T, Ogiwara I, Mazaki E, Yanagawa Y, Yamakawa K (2017) Nav1.2 is expressed in caudal ganglionic eminence-derived disinhibitory interneurons: Mutually exclusive distributions of Nav1.1 and Nav1.2. Biochemical and Biophysical Research Communications 491:1070-1076.
